# Filamentous calcareous alga provides substrate for coral-competitive macroalgae in the degraded lagoon of Dongsha Atoll, Taiwan

**DOI:** 10.1371/journal.pone.0200864

**Published:** 2019-05-16

**Authors:** Carolin Nieder, Chen-Pan Liao, Chaolun Allen Chen, Shao-Lun Liu

**Affiliations:** 1 Leigh Marine Laboratory, Institute of Marine Science, University of Auckland, Leigh, New Zealand; 2 Department of Life Science & Center for Ecology and Environment, Tunghai University, Taichung, Taiwan; 3 Department of Biology, National Museum of Natural Science, Taichung, Taiwan; 4 Biodiversity Research Center, Academia Sinica, Taipei, Taiwan; 5 Institute of Oceanography, National Taiwan University, Taipei, Taiwan; 6 Department of Life Science, National Taiwan Normal University, Taipei, Taiwan; University of Bremen, GERMANY

## Abstract

**Background:**

The chemically-rich seaweed *Galaxaura* is not only highly competitive with corals, but also provides substrate for other macroalgae. Its ecology and associated epiphytes remain largely unexplored. To fill this knowledge gap, we undertook an ecological assessment to explore the spatial variation, temporal dynamics, and diversity of epiphytic macroalgae of *Galaxaura divaricata* on patch reefs in the lagoon of Dongsha Atoll, a shallow coral reef ecosystem in the northern South China Sea that has been repeatedly impacted by mass coral bleaching events.

**Methods:**

Twelve spatially independent patch reefs in the Dongsha lagoon were first surveyed to assess benthic composition in April 2016, and then revisited to determine *G*. *divaricata* cover in September 2017, with one additional *Galaxaura*-dominated reef (site 9). Four surveys over a period of 17 months were then carried out on a degraded patch reef site to assess the temporal variation in *G*. *divaricata* cover. Epiphytic macroalgae associated with *G*. *divaricata* were quantified and identified through the aid of DNA barcoding at this degraded site.

**Results:**

Patch reefs in the Dongsha lagoon were degraded, exhibiting relatively low coral cover (5–43%), but high proportions of macroalgae (13–58%) and other substrate (rubble and dead corals; 23–69%). The distribution of *G*. *divaricata* was heterogeneous across the lagoon, with highest abundance (16–41%) in the southeast area. Temporal surveys showed consistently high covers (mean ± SD = 16.9 ± 1.21%) of *G*. *divaricata* for 17 months. Additional photographic evidence suggested that overgrowth of *G*. *divaricata* can persist for 3.5 years. Yet, *G*. *divaricata* provides substrate to other macroalgae (e.g., *Lobophora* sp.) that also limit the growth of corals.

**Conclusions:**

Our study demonstrates that an allelopathic seaweed, such as *G*. *divaricata*, can overgrow degraded coral reefs for extended periods of time. By providing habitat for other harmful macroalgae, a prolonged *Galaxaura* overgrowth could further enhance the spread of macroalgae, and strengthen negative feedback loops, decreasing the recovery potential of degraded reefs.

## Introduction

Coral-macroalgae competition is a natural ecological process on coral reefs [1]. However, anthropogenic disturbances, e.g., climate change, overfishing, and pollution, have intensified space competition between macroalgae and corals and in turn led to a phase shift from a coral-dominated to a macroalgae-dominated ecosystem [2]. The recovery of corals on degraded reefs is strongly influenced by the type of dominant macroalgae, i.e., allelopathic versus non-allelopathic [3,4]. Allelopathic macroalgae produce lipid-soluble secondary metabolites, e.g., loliolide derivatives or terpenes that are poisonous to corals (known as allelochemicals). Such allelochemicals are capable of bleaching and killing coral tissue [5], decreasing the photosynthetic efficiency of zooxanthellae [6], and altering the coral microbiome, ultimately decreasing coral health [7,8]. Allelopathic macroalgae are considered most detrimental for the resilience of coral reefs [9], as these types may perpetuate their dominance by deterring coral larval settlement, and inhibiting the growth and survival of juvenile recruits, key processes of coral reef recovery [10–12].

The red upright calcifying seaweed *Galaxaura* is known to be highly allelopathic against corals [13]. Life history of the genus *Galaxaura* can be grouped into two morphotypes, a smooth and a filamentous type. The latter is characterized by hairy branches that are covered with fine assimilatory filaments [14]. Extracts of the lipid-soluble secondary metabolites of *G*. *filamentosa* were shown to cause bleaching and death of coral tissue [13,15], and deterred coral larvae from settling [16]. It has thus been suggested that high abundance of *Galaxaura* on degraded reefs can inhibit the recovery of coral cover [4,9,16].

The filamentous morphotype of *G*. *divaricata*, is widely distributed in subtropical and tropical reef areas in the Pacific Ocean [17]. Filamentous *G*. *divaricata* is also common on coral reefs in the shallow lagoon of Dongsha Atoll [18]. Dongsha Atoll is the only large (> 500 km^2^) coral reef atoll in the northern South China Sea and represents a highly valuable hot-spot for marine biodiversity in this region [19]. A catastrophic mass bleaching in 1998 and reoccurring bleaching events thereafter have, however, caused severe mass mortalities of corals in the Dongsha lagoon, followed by a marked increase of macroalgae [20,21]. To date, little is known about state of recovery and dominant macroalgae in Dongsha lagoon patch reefs. The proliferation of *G*. *divaricata* on degraded reefs in the lagoon of Dongsha Atoll was first uncovered during a systematic macroalgae sampling expedition in February 2014 [18]. Similar to some well-documented macroalgae (e.g., crustose *Lobophora* or canopy-forming *Sargassum* and *Turbinaria*) that provide habitat for epiphytes [22–24] we observed that *G*. *divaricata* was also highly populated by epiphytic macroalgae. The dense epiphytic community associated with *G*. *divaricata* might indicate a previously unappreciated role of *Galaxaura* as a habitat forming seaweed.

The goals of this study were to 1) assess the benthic composition of lagoon patch reefs, 2) document the spatial distribution of *G*. *divaricata* on patch reefs in the lagoon, 3) monitor changes of *G*. *divaricata* percent cover over time, and 4) quantify and identify the epiphytic macroalgae associated with *G*. *divaricata*. Understanding the dynamics *G*. *divaricata* and its role in providing new habitat for other macroalgae is important because the epiphytic community on *G*. *divaricata* could enhance macroalgae biodiversity on the reef, or provide trophic support for herbivores, while a facilitation of allelopathic algal types would decrease the resilience of coral reefs.

## Materials and methods

### Ethics statement

The ecological assessments and sample collections in this study were conducted with permissions of the Dongsha Atoll National Park.

### Site description

**Fig 1:**
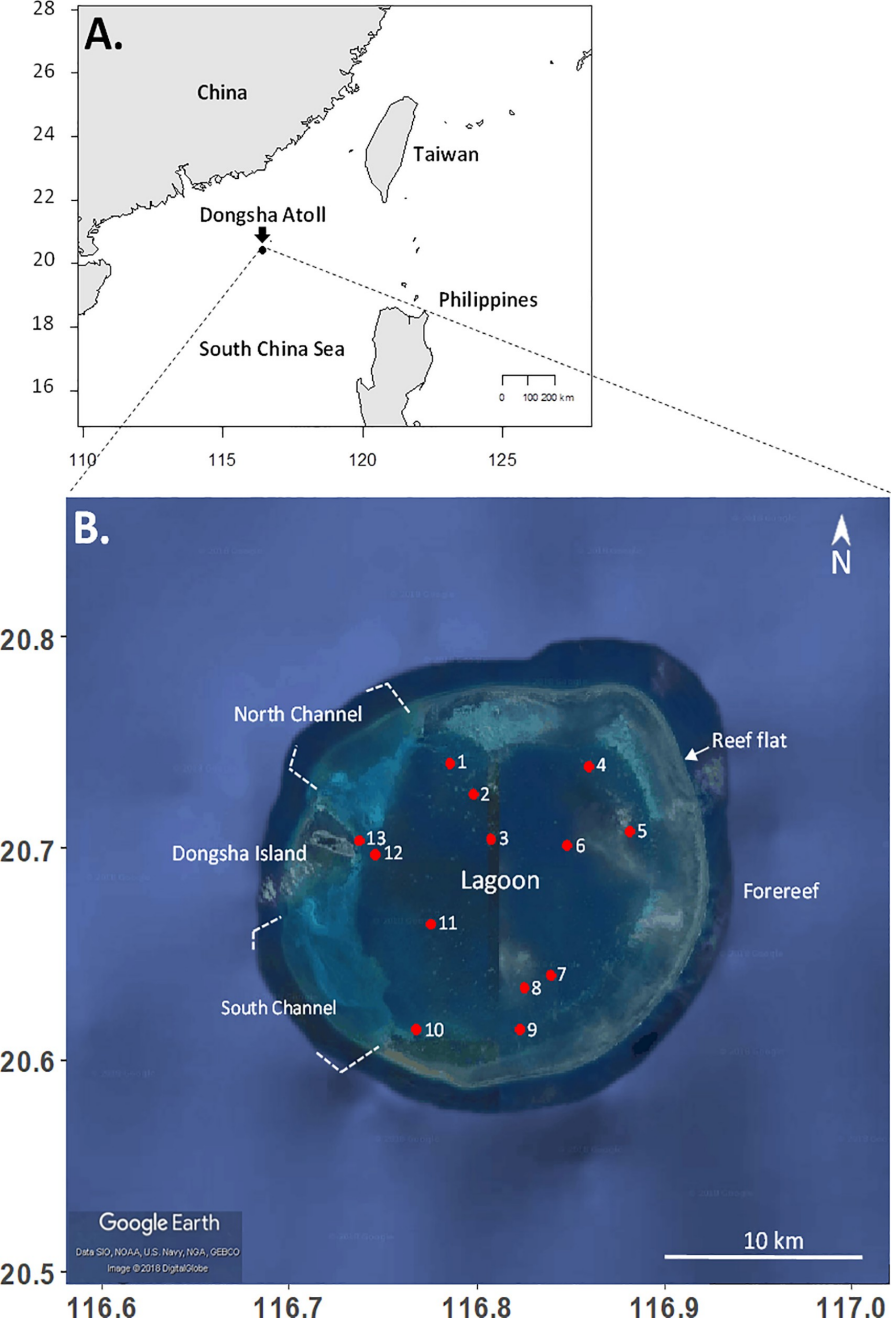
Study site. A) Geographical location of Dongsha Atoll in the northern South China Sea. B) Lagoon patch reef sites surveyed in this study.

This study was conducted from April in 2016 to September 2017 in the lagoon of Dongsha Atoll (also known as Pratas Island; 20^o^40’43” N, 116^o^42’54” E), which is an isolated coral reef atoll in the northern South China Sea. The atoll covers an area of approximately 500 km^2^ and is situated 450 km southwest from the coast of Taiwan and 350 km southeast from Hong Kong (Fig 1A). The climate is seasonal and varies between a northeast monsoon winter (October-April) and southwest monsoon summer (May-September) [25]. Field work during the northeast winter monsoon is often restricted due to local weather conditions. The ring-shaped reef flat encircles a large lagoon with seagrass beds and hundreds of coral patch reefs [26]. Channels at the north and south of the small islet (1.74km^2^) interrupt the reef flat and allow for water exchange between the lagoon and the open ocean. The semi-closed lagoon is about 20 km wide with a maximum depth of 16 m near the center [20]. The lagoon patch reefs are structured into reef tops (1–5 m depth) and reef slopes (5–12 m depth), and provide important habitat and sheltered nursery grounds for numerous marine organisms, such as green sea turtles and coral reef fish, including rays and sharks [26]. For background information the lagoon water temperature was measured at each survey site, every 30 min from March 2016 to September 2017 using HOBO Pendant Temperature/Light 8K Data Loggers (UA-002-08, Onset Computer Corporation, USA). Water temperatures were highest during the summer monsoon, averaging 30.1°C, and lowest during the winter monsoon, averaging 24.8°C. Maximum temperatures from July to August reached 34°C on reef tops and 32.7°C on reef slopes.

### Spatial variation in benthic composition and *G*. *divaricata* cover of lagoon patch reefs

To assess the benthic composition of patch reefs in the lagoon of Dongsha Atoll, 12 spatially independent reefs were initially surveyed with SCUBA in April 2016 (Fig 1B and S1 Table). A 45-m transect was laid out across each reef area: reef top (2 m depth) and reef slope (10 m depth). The two transects were 10–20 m apart from each other. The percent cover of corals, total macroalgae (MA; all upright growing (including *G*. *divaricata*) and crustose non-coralline algae, and low growing, filamentous turf algae [27]), crustose coralline algae (CCA), and other substrate was estimated using a 35 cm x 50 cm PVC sapling frame [28]. Other substrate mainly consisted of dead coral, rubble, and rocks covered with sediments. Estimates were done *in-situ* at 1 m intervals, for a total of 45 sampling frames analyzed per transect. The 12 sites were revisited in September 2017 to estimate the percent cover of *G*. *divaricata* and corals only, using the same survey method described above. An additional patch reef (site 9) was included, as this site was historically shown to be dominated by *G*. *divaricata* based on photo evidence, resulting in a total of 13 survey sites (Fig 1B and S1 Table). The diameter of haphazardly selected *G*. *divaricata* thalli were measured *in situ* at each site and classified as small (1–5 cm diameter), medium (>5–15 cm diam.), and large (>15–30 cm diam.).

### Temporal variation in *G*. *divaricata* cover

To assess variations in *G*. *divaricata* cover over time, we selected the slope of a degraded patch reef (site 7) that was considerably overgrown by *G*. *divaricata* (14–18%) and had relatively low coral cover (13–19%). Percent cover of *G*. *divaricata* was estimated in April 2016 (the last month of the winter monsoon season), three times during the summer monsoon season (July and September 2016), and in September 2017, spanning a period of 17 months. Coral cover was also monitored during each survey to evaluate whether or not corals recovered over the course of this study. During each survey 45 photographs of the reef bottom were taken with an Olympus Stylus-TOUGH TG4 digital camera (25–100 lens, 35mm equivalent) mounted at 64 cm above the bottom onto a movable PVC-sampling frame (35 cm x 50 cm). A 45-m transect line was laid out along a marked trajectory to keep the survey area consistent among time points. For each survey the sampling frame was moved along the transect line and pictures were taken at every meter mark. Cover estimates were obtained from photographs using ImageJ software (version 1.52a) with a superimposed 10 x 10 reference grid, where 1 square represented 1% of the total grid area.

### Epiphytic macroalgae associated with *G*. *divaricata*

In September 2017, thirty thalli of *G*. *divaricata* were collected from a degraded reef (site 7) with relatively high percent cover of *G*. *divaricata* (14–18%). *G*. *divaricata* thalli were haphazardly collected across the reef slope along a 45-m transect at 5 m depth. Epiphytic macroalgae were removed and identified to the closest identifiable taxonomic unit, using either the Dongsha seaweed guide book [18] or DNA barcoding. The presence and absence of each taxonomic unit was recorded, and the occurrence frequency (*f*) was calculated as follows: *f* = *c*_*i*_/*n*, where *c*_*i*_ stands for the number of thalli that have the epiphyte taxonomic unit *i*, and *n* = 30, the total number of thalli analyzed. For DNA barcoding, macroalgae samples were preserved in silica gel after collection, and the total genomic DNA of samples was extracted with Quick-DNA Plant/Seed Miniprep Kit (Zymo Research Co., USA). Primers for the plastid gene specific amplifications were used as follows: *rbc*L *F7/R753* for red algae [29], *rbc*L *F68/R708* for brown algae [30], and *tufA F210/R1062* for green algae [31]. The newly generated sequences were deposited in GenBank and searched using BLASTn against the GenBank database (S2 and S3 Tables). Sequence similarities of >98% were considered for species identification.

### Statistical analysis

First, we explored spatial variation in benthic composition using nonmetric multidimensional scaling (NMDS) in the R package ‘vegan’ (version 2.5–4) [32]. Prior to this analysis, benthic cover was averaged within reef areas at each site and standardized using the Hellinger transformation. We then applied Spearman's Mantel tests to determine whether spatial distance between surveyed areas correlated with Bray-Curtis dissimilarities for both reef tops and reef slopes.

Next, we examined the effects of two independent variables (i.e., area and site) and their interaction on benthic composition through space and time using linear models with the integration of Bayesian Markov Chain Monte Carlo (MCMC) methods in the R package ‘brms’ (version 2.7.0) [33]. The prior specification of the Bayesian MCMC analysis is provided in S1 Text.

Prior to the analyses, the percent cover from each quadrat of transects was transformed using the logit transformation,

$${y=Log}_{e}\left(\frac{p}{1-p}\right),$$

where *p* stands for cover ratio. If the observed data matched 0% or 100%, the percent covers were proportionally remapped to 0.05–99.95%. Four different linear models were compared:

$$M_{1}:y_{ijk}=\mu_{ij}+\alpha {\left(area\right)}_{j}+{\left(site\right)}_{i}\sigma_{i}+(area)(site{)}_{i}\delta_{i}+\varepsilon_{ijk},$$

$$M_{2}:y_{ijk}=\mu_{ij}+(site{)}_{i}\sigma_{i}+(area){\left(site\right)}_{i}\delta_{i}+\varepsilon_{ijk},$$

$${M_{3}:y}_{ijk}=\mu_{ij}+(site{)}_{i}\sigma_{i}+\varepsilon_{ijk},\;and$$

$${M_{4}:y}_{ijk}=\mu_{ij}+\varepsilon_{ijk},$$

where *y* denotes the logit-transformed percent cover *p* of a specific benthic category on *k*^th^ quadrat in area *j* within site *i*, *α* denotes the fixed effect of area *j*, *σ* denotes the random intercept by site, and *δ* denotes the random slope against areas by site. The best model was selected based on Bayes factors. Overall, a multivariate mixed-effect linear model best fit the percent cover of corals, macroalgae, CCA, and other substrate, whereas a mixed-effect linear model best fit the percent cover of *G*. *divaricata*. Similarly, to examine temporal variation in the percent cover of *G*. *divaricata* and corals at site 7, we applied the multivariate fixed-effect linear model, except with time rather than area as the fixed factor and no random factor. In both our spatial and temporal analyses, pair-wise Bayesian MCMC tests were conducted to examine which pairs of surveyed areas were significantly different from each other. The tests were considered statistically significant if the effect size was greater than 0.2 (Cohen’s *d* > 0.2).

## Results

### Benthic composition

**Fig 2:**
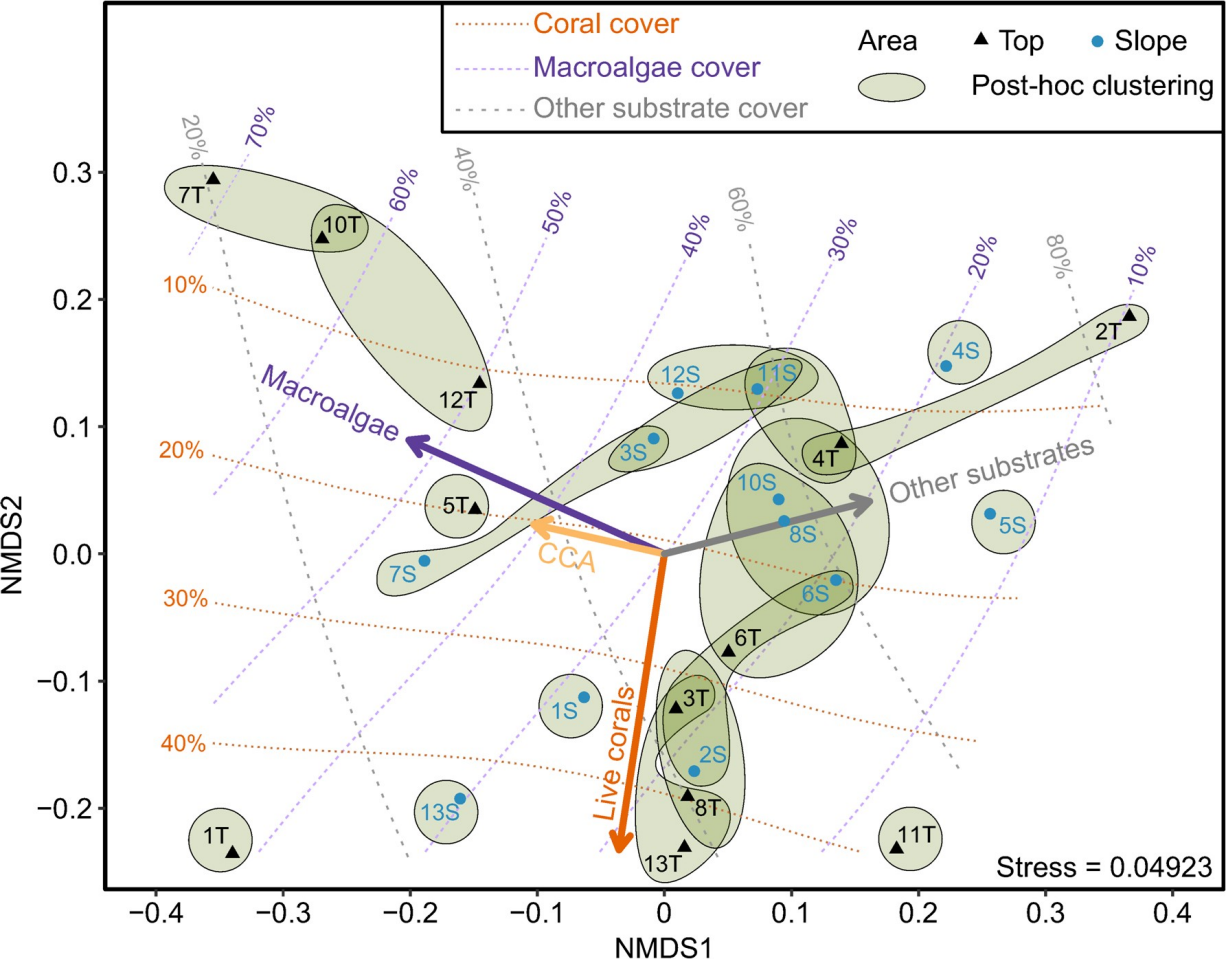
Spatial variation in benthic categories in the Dongsha lagoon. Two-dimensional nonmetric multidimensional scaling (NMDS) of average percent cover of corals, total macroalgae (upright/crustose non-coralline seaweeds and turf), crustose coralline algae (CCA) and other substrate among 24 groups (12 sites × 2 area types). Thick arrows represent the vectors of weighted average scores of benthic categories. Green loops enclose groups with statistically similar composition by clustering analysis based on the Bayesian post-hoc analyses (posterior Cohen’s *d* > 0.2); a loop enclosing only one group indicates that its composition is significantly different from all other groups. Dashed lines indicate the contours of percent cover projected onto two-dimensional NMDS space.

Our NMDS analysis revealed that macroalgae cover was highest (> 40%) on the reef top and reef slope of site 7 and on the reef tops of sites 1, 5, 10, and 12. Tops and slopes of sites 1 and 13 exhibited highest coral covers (Fig 2A and 2B; S1A Fig). Notably, the reef top of site 1 was the only area showing both higher cover of corals and macroalgae (Fig 2A; S1A Fig). Compared with corals and total macroalgae, we found that CCA cover was relatively low (< 3%) across sites. The average “other substrate” cover (mainly dead coral, rubble, and rocks) was generally high (> 40%) in over 50% of the surveyed areas. Our post-hoc tests showed that our 24 transects from reef top and slope across 12 sites could be grouped into 19 clusters (Fig 2A). Our Mantel test showed no correlation between spatial distances and benthic composition dissimilarities among sites in either reef top or slope (Mantel test; slope area, *r*_s_ = 0.152, *P* = 0.132; top area, *r*_s_ = 0.032, *P* = 0.590; S2 Fig).

Our linear model analyses showed that the area by site interaction effect had significant influence on the percent cover of corals, macroalgae, CCA, and other substrate (S1A Fig; BF > 150, BF of M_2_ over M_3_ in S4 Table). Our post-hoc analysis revealed a significant difference between top and slope in five sites for corals, seven sites for macroalgae and other substrate, and eight sites for CCA (S1A Fig). For instance, the interaction effect was evident in the percent cover of four different benthic categories (e.g., corals: top > slope in site 3 and slope > top in site 2; macroalgae and other substrate: top > slope in site 1 and slope > top in site 2; and CCA: with a much lower percent cover on the slope of site 4 compared to the slope of other sites) (S1A Fig). Overall, no clear spatial pattern was observed in benthic composition (S1B Fig).

### Spatial variation in *G*. *divaricata* cover

**Fig 3:**
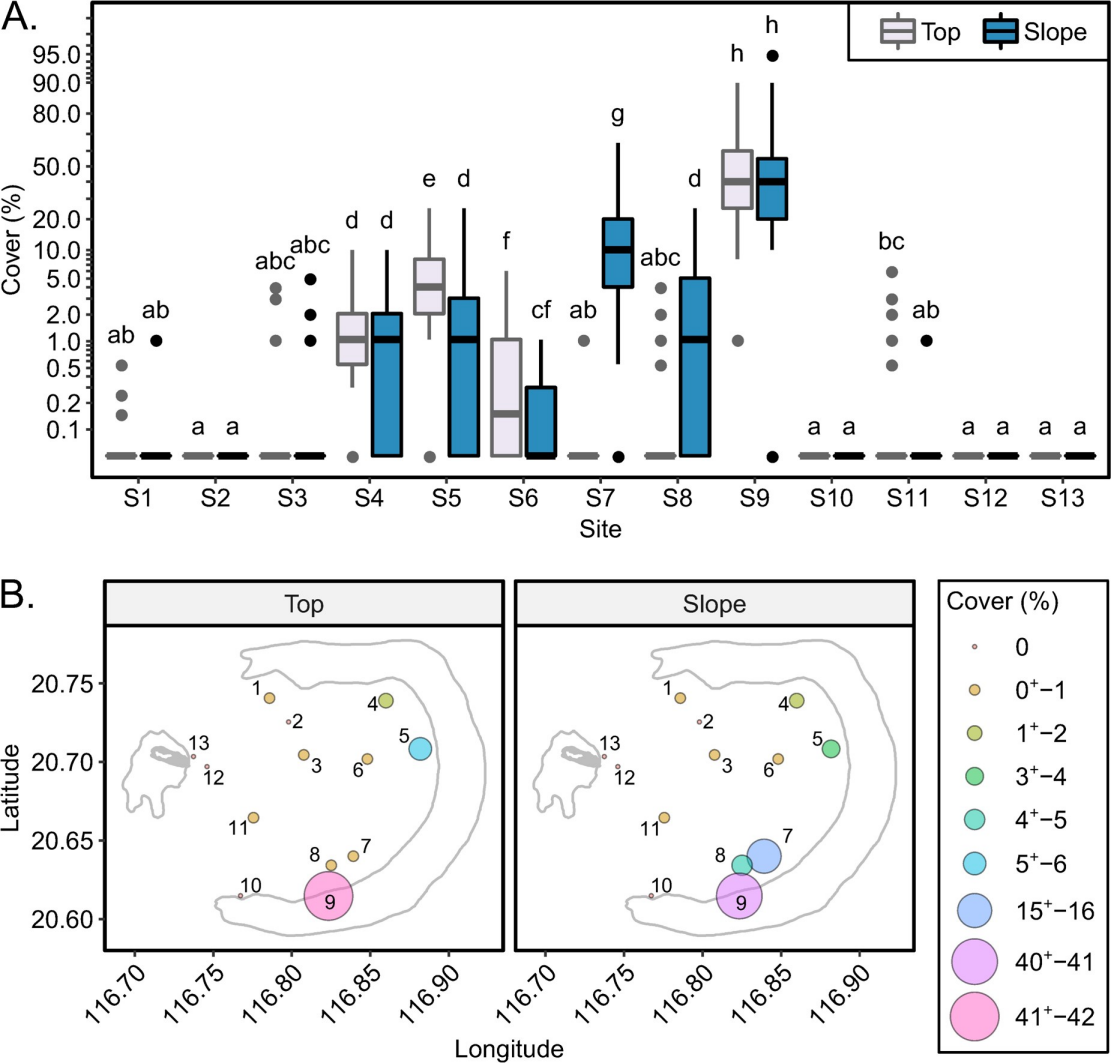
Spatial variation in *G*. *divaricata* in the Dongsha lagoon. (A) Boxplots showing variation in the percent cover of *G*. *divaricata* between reef top and slope among 13 sites. Labels denote the ranks of percent cover according to multiple comparisons among 26 transects. Labels that with different letters indicate a statistical difference (posterior Cohen’s *d* > 0.2) in the *G*. *divaricata* cover. (B) The average percent cover on the reef top and slope at each site are represented by circles with different colors and sizes.

The percent cover of *G*. *divaricata* among 13 sites was statistically significantly affected by the interaction between area (reef top and reef slope) and site (e.g., top > slope in site 5 and 6, and slope > top in site 7 and 8 in Fig 3A; BF > 150, BF of M_2_ over M_3_ in S5 Table). Our post-hoc tests showed that *G*. *divaricata* cover was not at all similar among the 26 transects (Fig 3). *G*. *divaricata* was most abundant on survey sites in the southeast lagoon, e.g., in site 9 (41%) and on the slope of site 7 (16%) (Fig 3A and 3B). Patch reefs in the northeast lagoon exhibited much lower cover of *G*. *divaricata* (range: 0.21–5.7%) (Fig 3A and 3B and S6 Table). Survey sites in the south, center, west, and north of the lagoon were characterized by the lowest cover of *G*. *divaricata* (range: 0–1.4%; Fig 3B and S6 Table).

During our survey, we observed that the thallus shape and size of *G*. *divaricata* varied across sites (S3 Fig). Small ball-shaped or slender thalli were dominant on patch reefs in the northeast lagoon, while medium ball-shaped and large, carpet-like thalli were exclusively present in the southeast lagoon. Our DNA barcoding analyses confirmed that all samples across sites were 100% identical in their *rbc*L sequences, indicative of conspecificity (S3 Table).

### Temporal dynamics of *G*. *divaricata* cover

**Fig 4:**
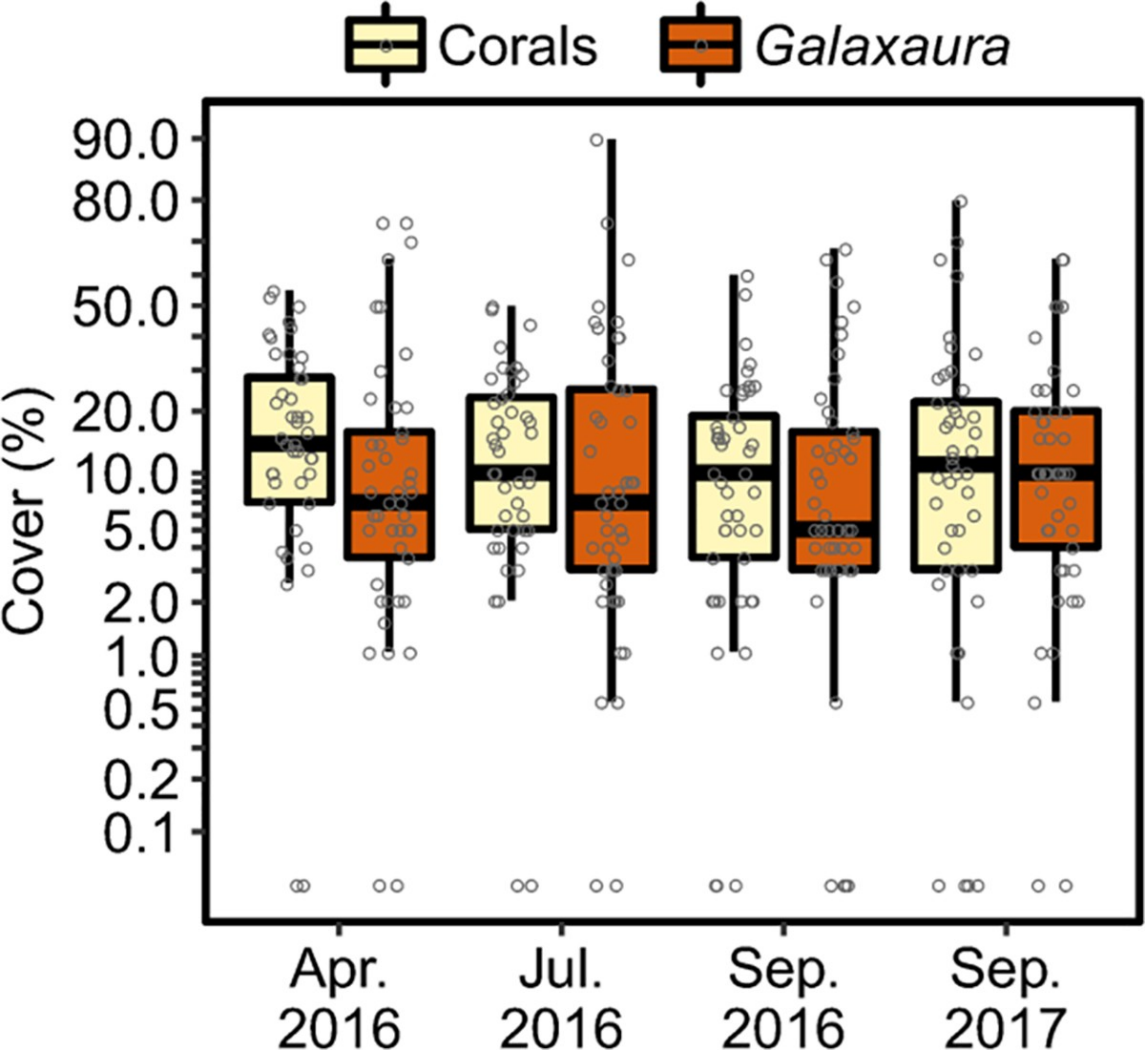
Temporal variation in *G*. *divaricata* on a degraded patch reef in the southeast Dongsha lagoon. Boxplots showing variation in the percent cover of two major benthic categories (corals and *G*. *divaricata*) among four time points over a period of 17 months (about 5 m depth at site 7).

**Fig 5:**
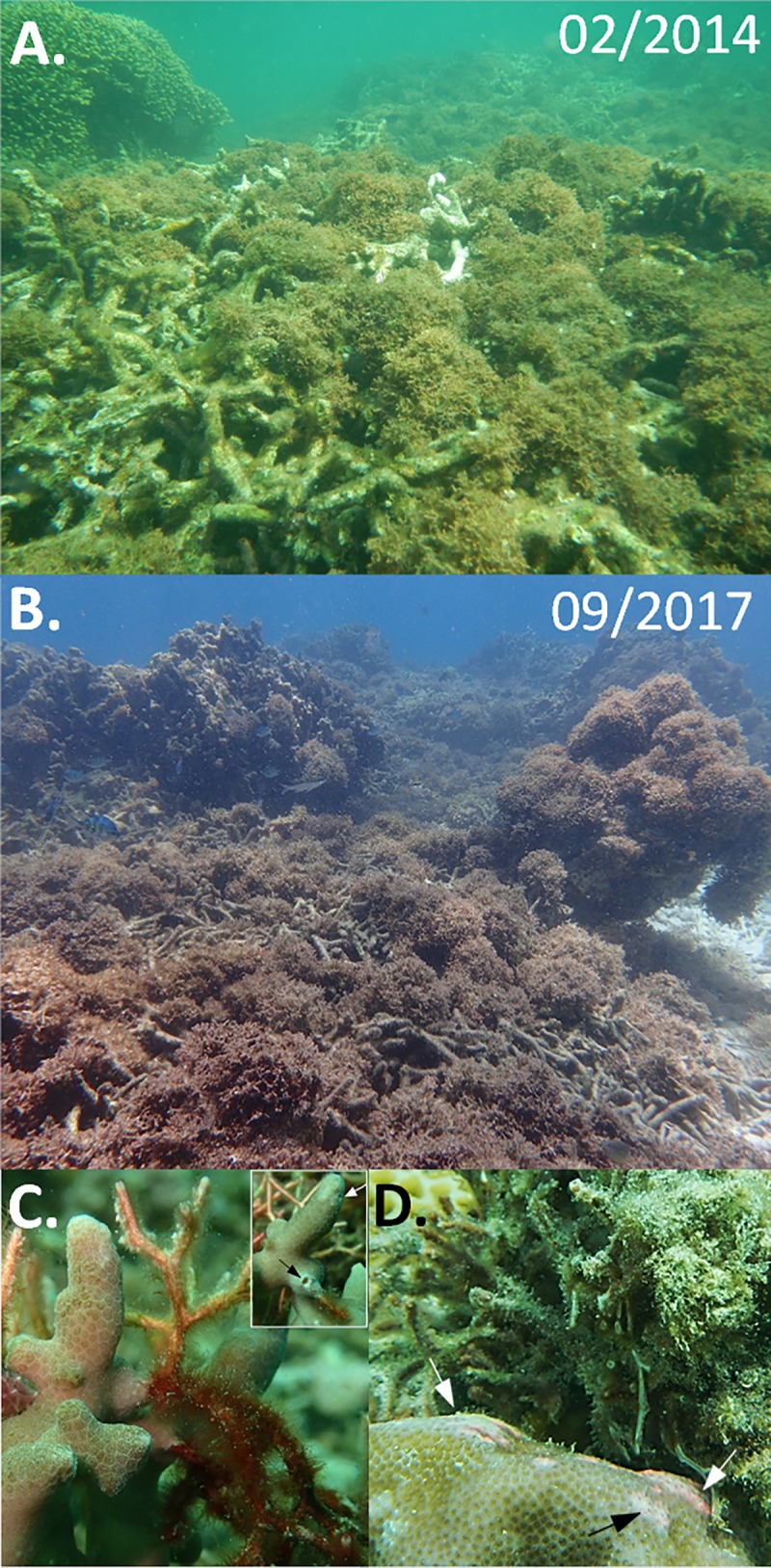
Observational photo-evidence of a prolonged *Galaxaura* overgrowth. A-B) A degraded patch reef in the southeast lagoon of Dongsha Atoll has been overgrown by *G*. *divaricata* for at last 3.5 years (3–5 m depth at site 9). Photos were taken in A) February 2014, with water temperature = 22.5°C; and B) in September 2017 with water temperature = 29°C. The holdfast of *G*. *divaricata* penetrates a branching *Porites* coral (*P*. *cylindrica*), creating small holes (inset). D) Coral (*P*. *solida*) tissue discoloration and bleaching (arrows) following direct contact with *G*. *divaricata*, potentially caused by allelopathic chemicals.

Our temporal survey at a *Galaxaura*-dominated reef (slope of site 7) revealed that the percent covers of *G*. *divaricata* and corals were similar among 4 time points (April 2016, July 2016, September 2016, and September 2017). The main effect (time points) contributed only 2% and 2.7% partial *R*^2^ when fitting *G*. *divaricata* and coral covers, respectively, showing that both covers did not significantly change over a period of 17 months (BF < 0.001; ΔLOOIC = −9.36; Fig 4). Throughout the study, the mean *G*. *divaricata* cover remained relatively high (16.45 ± 1.17%), while mean coral cover was low (15.91 ± 0.6%). In addition, we provide photo-evidence from an additional patch reef (site 9, 3–5 m) overgrown by *G*. *divaricata*. Photographs of the site were taken in February 2014 and in September 2017, showing that the same *G*. *divaricata* overgrowth was present after 3.5 years (Fig 5A and 5B). *G*. *divaricata* frequently grew on live corals, where the holdfast penetrated the calcium-carbonate structure, creating a strong attachment to the corals (Fig 5C). In several cases we observed a fluorescent pink discoloration and bleaching of the coral tissue at the contact zone with *G*. *divaricata*, strongly indicative of allelopathic inhibition by *G*. *divaricata* (Fig 5D).

### Epiphytic macroalgae associated with *G*. *divaricata*

**Fig 6:**
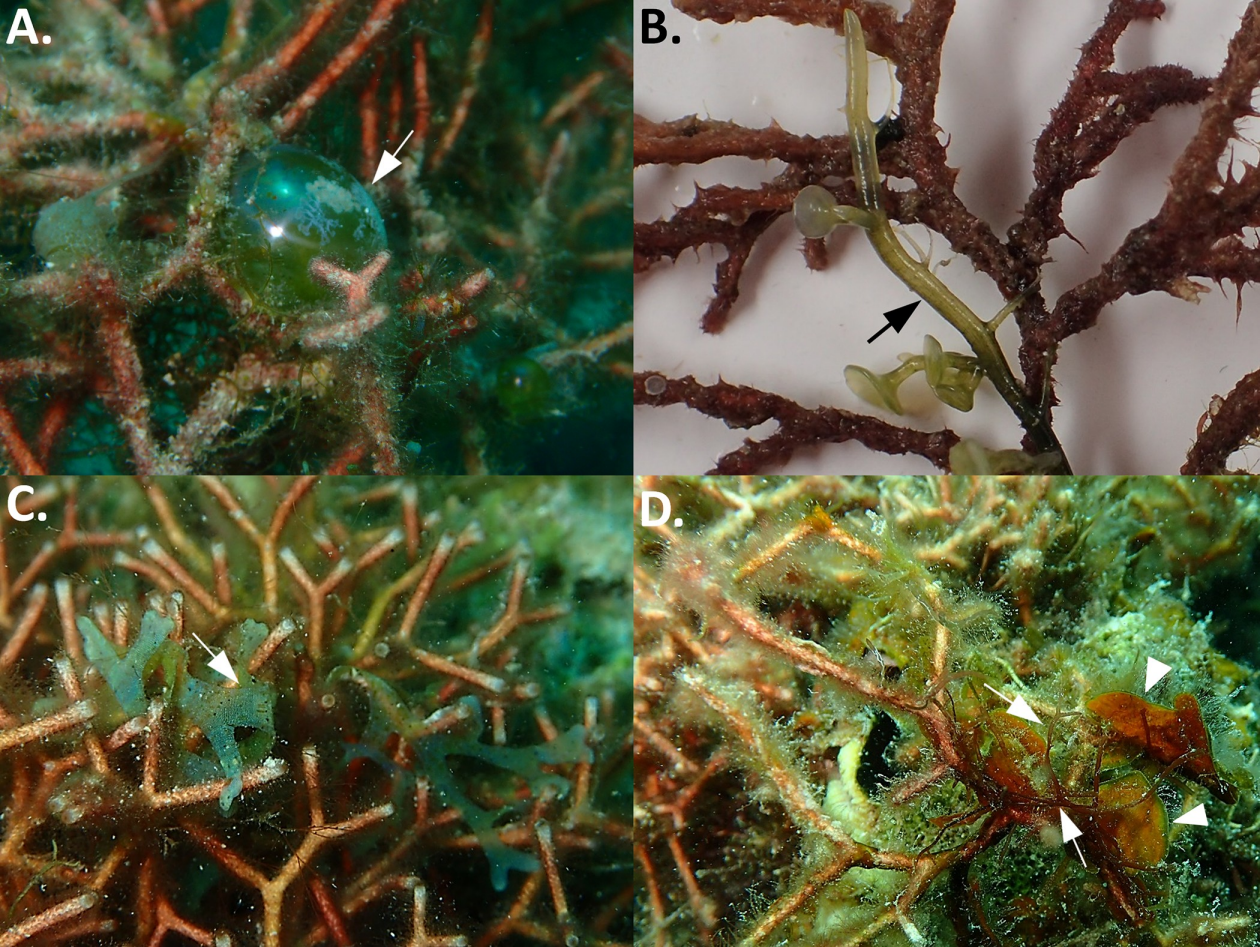
Examples showing epiphytic macroalgae that frequently grow on *G*. *divaricata*. A) *Valonia ventricosa*, B) *Caulerpa chemnitzia*, C) *Dictyota* sp., D) *Lobophora* sp. (arrowhead), and *Hypnea caespitosa* (arrow).

**Fig 7:**
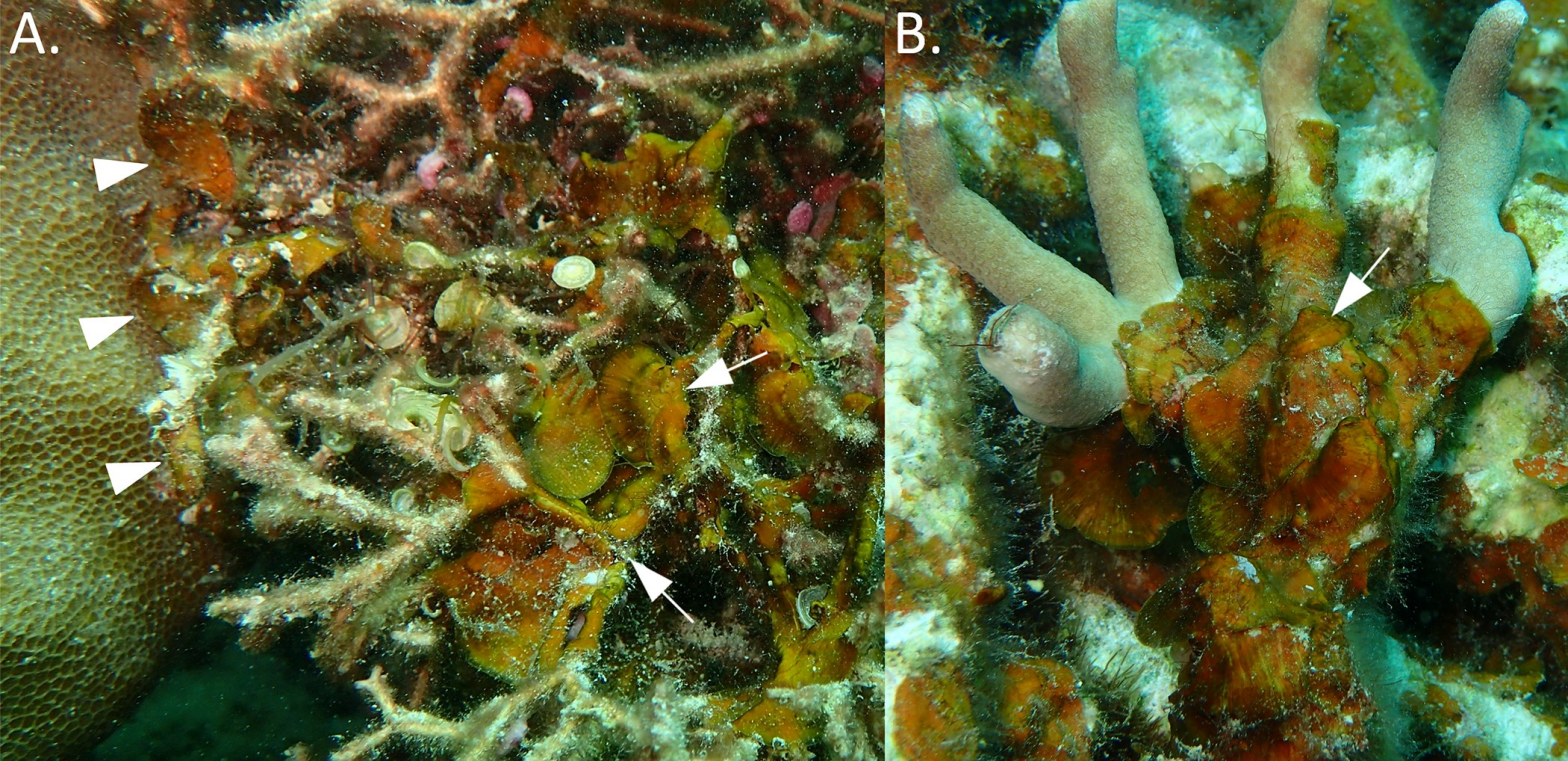
Coral overgrowth by *Lobophora* sp28. A) Example showing *Lobophora* sp28 growing on *Galaxaura divaricata* (arrows), and in contact with coral (*Porites solida*) (arrowheads). B) Coral overgrowth (*Porites cylindrica* in this case) by *Lobophora* sp28 is widespread in the shallow lagoon of Dongsha Atoll.

We identified 21 taxonomic groups of macroalgae, including macroscopic filamentous cyanobacteria, in association with *G*. *divaricata* (Table 1 and S2 Table). Among these, 15 were identified to the species level, with seven species of red algae, three species of brown, and five species of green algae (Table 1 and S2 Table). The most common green macroalgae associated with *G*. *divaricata* were *Derbesia marina* (occurrence frequency: 37%) (Fig 6A), *Caulerpa chemnitzia* (27%) (Fig 6B), and *Boodlea composita* (20%). The most common brown macroalgae associated with *G*. *divaricata* were the brown algae *Lobophora* sp. (as *Lobophora* sp28 in [34]) (57%), *Padina* sp. (as *Padina* sp5 in [35]) (53%), and *Dictyota bartayresiana* (30%) (Fig 6C). The most common red macroalgae associated with *G*. *divaricata* were *Hypnea caespitosa* (100%) (Fig 6D), *Coelothrix irregularis* (87%), *Ceramium dawsoniia* (43%). Lastly, epiphytic macroscopic cyanobacteria (> 1cm in height) had an occurrence frequency of 17%. Among these epiphytic macroalgae we observed that L*obophora* (identified as *Lobophora* sp28; S2 Table) was also found to frequently overgrow corals in the Dongsha lagoon (Fig 7 and S4 Fig).

**Table 1:** Occurrence frequency (%) of epiphytic macroalgae on *Galaxaura divaricata* from the slope of site 7.

Epiphyte taxon	Phylum	Occurrence frequency (%)
*Acanthophora spicifera*^a^	Red	3
*Ceramium dawsonii*_(MH048927)_^b^	Red	43
*Coelothrix irregularis*_(MH048928)_	Red	87
*Dichotomaria obtusata*	Red	3
Gelidiales	Red	27
*Gracilaria* spp.	Red	7
*Hypnea caespitosa*_(MH048929, MH048930, MH048931)_	Red	100
*Hypnea* sp. _(MH048932)_	Red	30
*Laurencia dendroidea*	Red	13
*Laurencia* spp.	Red	20
*Dictyota bartayresiana*	Brown	30
*Dictyota* spp.	Brown	13
*Lobophora* sp28^c^_(MH048934, MH048935, MH048936, MH048937)_	Brown	57
*Padina* sp5^d^_(MH048933)_	Brown	53
*Sargassum* spp.	Brown	3
*Boodlea composita*	Green	20
*Caulerpa chemnitzia*_(MH048959)_	Green	27
*Derbesia marina*	Green	37
*Phyllodictyon anastomosans*	Green	10
*Valonia ventricosa*	Green	10
Cyanobacteria (filamentous > 1cm)	Cyanobacteria	17

^a^Identification of species and taxonomic groups according to [18].

^b^GenBank number in parentheses based on species identifications through DNA barcoding.

^c^Denomination according to [34].

^d^Denomination according to [35].

## Discussion

Our study shows that most patch reefs in the lagoon of Dongsha Atoll are degraded. Many of the lagoon patch reefs (ca. 63% of the surveyed areas) exhibit relatively low coral cover (< 30%) and high proportions of macroalgae, dead corals, and rubble, all of which are signs of reef degradation [36]. This is consistent with previous surveys that reported degraded conditions of lagoon patch reefs at Dongsha Atoll [37,38]. The filamentous form of *Galaxaura divaricata* showed highest abundance in the southeast lagoon. *Galaxaura* overgrowth was also observed in other locations in the southeast lagoon in previous surveys (Keryea Soong, personal communication; our own macroalgae inventory in 2012). The exact causes for this localized condition are not well understood. However, a potential explanation may be that the southeast lagoon is sheltered by a 2 km-wide reef flat, harboring very shallow (1–5 m) and calm waters that may provide suitable growth conditions for *G*. *divaricata*. The proliferation of macroalgae is likely the consequence of an initial coral decline [39,40]. The synergistic effects of thermal stress, overfishing, and typhoon damage may have caused the decline of the once pristine corals in the Dongsha lagoon, followed by a proliferation of *G*. *divaricata* and other macroalgae. Thermal stress on corals has increased over the past decades, with waters surrounding Dongsha Atoll warming at a faster rate than other areas of the South China Sea [37,41,42]. Recurrent bleaching events have caused high coral mortality and eradicated thermo-sensitive coral genera from the lagoon [43]. Overfishing and the extensive use of dynamite and cyanide, prior to the establishment of the Dongsha Atoll National Park in 2007 reduced fish, and destroyed large areas of coral framework [20,44]. Insufficient grazing by fish after disturbance can lead to the establishment and full outgrowth of macroalgae beyond their early stages [45]. *Galaxaura* is known to be largely unpalatable for various herbivorous fishes due to its calcareous thallus and low nutritional content [46–48]. Local herbivorous fish population in the Dongsha lagoon may not be effective to control the outgrowth of *Galaxaura* in certain areas.

Semi-closed lagoons are highly vulnerable to eutrophication and hypoxia, especially under the backdrop of climate change [49,50]. Reoccurring events of hypoxia during hot summers in 2014 and 2015 have caused substantial mass-die offs of the coral associated fauna and flora in the Dongsha lagoon [51]. Particularly, densities of macroinvertebrates, including echinoids, sea cucumbers, lobsters, and giant clams are extremely low (S7 Table). *Galaxaura* appears to be well adapted to hypoxic conditions. For instance, *G*. *filamentosa* was one of the few algae to proliferate after a mass-die off caused by hypoxia in an atoll lagoon in French Polynesia [52].

Although the filamentous *G*. *divaricata* is a common allelopathic seaweed in subtropical and tropical waters, it has never been reported as a nuisance in overgrowing coral reefs. Our observations are the first to report a prolonged *G*. *divaricat*a overgrowth in degraded coral reefs. For instance, the *G*. *divaricata* cover was equally high on a degraded reef after 17-months. We further provide photo-evidence from another patch reef showing that the same *G*. *divaricata* overgrowth was present to a similar extend after 3.5 years. The photos clearly show that *G*. *divaricata* dominated the reef substrate of the site in both, the cooler northeast monsoon (winter) season (Fig 5A, water temperature: 22.5°C), and the warmer southwest monsoon (summer) season (Fig 5B, water temperature: 29°C). Due to challenging weather conditions, we were only able to conduct our quantitative temporal survey in April, the last month of the winter season, and therefore we cannot rule out potential variations in *G*. *divaricata* cover over the full length of that season. Expanding temporal surveys in the future will be worth of doing to confirm the long-term persistence of *G*. *divaricata* overgrowth.

It is important to note that only two out of 13 survey sites showed substantial overgrowth by *G*. *divaricata*. Thus, the dominance of *G*. *divaricata* is not a generalized condition across the entire lagoon of Dongsha Atoll. Rather, it represents a much localized condition found at certain sites in the southeast lagoon.

High abundance of filamentous *G*. *divaricata* may have profound implications for the recovery potential of those patch reefs experiencing a prolonged *Galaxaura* overgrowth in the lagoon of Dongsha Atoll. Owning to its allelopathic effects on corals long-standing canopies of *G*. *divaricata* are likely to hamper coral recruitment ultimately preventing coral recovery [16,53]. As a caveat of this study, it is important to note that we did not attempt to isolate and identify allelopathic chemicals in *G*. *divaricata*. But, previous studies have identified lipid-soluble terpenoid compounds from filamentous *Galaxaura* cell extracts as allelochemicals that were capable of bleaching and killing coral tissue [13]. It is also known that *Galaxaura* can change the chemical microclimate on degraded reefs with adverse effects on fish feeding behavior [4]. For instance, butterflyfish and other corallivores avoid corals in close association with *Galaxaura*, making it potentially difficult for these trophic guilds to find food [54,55]. Unlike other calcifying algae such as coralline algae, *Galaxaura* does not stabilize the reef matrix. Thus, a prolonged *Galaxaura* overgrowth may contribute to the erosion and flattening of the reef structure, which negatively impacts biodiversity, and trophic support for coral associated organisms [56].

The filamentous *G*. *divaricata* is used as habitat by a variety of macroalgae. The availability of new habitat for epiphytic macroalgae provided by a prolonged *Galaxaura* overgrowth could have several implications for the ecology and recover potential of the reef. For instance, nutrient rich epiphytes could provide trophic support for herbivorous fishes and invertebrates, such as crustaceans and mollusks [24,57,58]. On the other hand, the association with the unpalatable *Galaxaura* may provide a refuge from herbivory for certain palatable algae [40,59,60], and facilitate their establishment on the reef, increasing macroalgae biodiversity [61]. The facilitation of harmful, allelopathic algal types could decrease the resilience and promote alternative stable states on coral reefs [62]. Some of the identified *G*. *divaricata* epiphytes, such as cyanobacteria [11], *Dictyota* [63], and *Lobophora* [10,64] are widely shown to overgrow corals after disturbance, and are known for their allelopathic inhibition of coral larvae recruitment. Here, we firstly report that an undescribed species *Lobophora* sp. (as *Lobophora* sp28 in [34]), the third most abundant macroalga on *G*. *divaricata*, overgrows and kills corals in the Dongsha lagoon through epizoism (Fig 7 and S2 Fig). Moreover, the microscopic filaments of *G*. *divaricata* may facilitate the attachment of macroalgae spores, while the calcified branches may provide structural support for fine, filamentous macroalgae. Considering that an increased substrate availability can promote macroalgae biomass on coral reefs, we hypothesize that, by providing a habitat for epiphytic macroalgae, *G*. *divaricata* may facilitate the diversity and abundance of macroalgae on degraded reefs. This study is merely observational and does not provide experimental evidence for the facilitation of macroalgae diversity and abundance by *G*. *divaricata*. However, the abovementioned hypotheses would be of great interest awaiting future validation.

## Conclusions

Our observations illustrated that the allelopathic and unpalatable filamentous seaweed, *Galaxaura divaricata*, can become dominant on degraded reefs in shallow, sheltered, and calm environments. We show that *G*. *divaricata* provides suitable substrate for a variety of macroalgae, further facilitating macroalgae growth and abundance on degraded reefs. Thus, a prolonged proliferation of *Galaxaura* could potentially enhance negative feedback loops, thereby perpetuating reef degradation. Several common epiphytic macroalgae on *Galaxaura* are allelopathic and known to frequently overgrow corals. Macroalgal assemblages, such as the *Galaxaura-*epiphyte system, warrant further investigation to better understand their ecological implications on the resilience of coral reefs, especially of shallow atoll lagoons. There are 439 listed coral reef atolls on earth; among them are 335 with semi-enclosed lagoons [65]. Atoll lagoons are highly productive and serve as valuable nursery habitat for marine life; however, they are most vulnerable to the effects of climate change [50,66]. Results from our study can be informative for the management and conservation of lagoons and shallow, inshore coral reef ecosystems, especially in the South China Sea and the Pacific Ocean, where filamentous *Galaxaura* is very common.

## Supporting information

S1 Fig **Percent cover (A) and spatial patterns (B) of corals, macroalgae, crustose coralline algae (CCA), and other substrate on reef top and slope across 13 sites.** Color and size of the circles are proportional to the percent cover.
(TIF)

S2 Fig Correlation between geographical distance and benthic composition dissimilarity among 12 sampling sites. Regression lines and 95% confidence bands were estimated by the locally weighted scatterplot smoothing (LOESS) method.
(TIF)

S3 Fig Various sizes and thallus shapes of Galaxaura divaricata from different locations in the lagoon of Dongsha Atoll. A-B) Small, ball-shaped thalli, and C-D) small, slender thalli were dominant on patch reefs in the north and northeast lagoon. E) Medium, ball-shaped thalli, and F) large carpet-like thalli were exclusively present in the southeast section of the lagoon.
(TIF)

S4 Fig Coral overgrowth by Lobophora sp28. A) Coral overgrowth (*Porites cylindrica* in this case) by *Lobophora* sp28 is widely spread in the lagoon of Dongsha Atoll. B) The same coral showing dead tissue (arrow) after the removal of the algae.
(TIF)

S1 Table GPS coordinates of patch reef survey sites in the lagoon of Dongsha Atoll, South China Sea (Taiwan). (DOCX)

S2 Table Information and Genbank numbers of macroalgae samples used for DNA barcoding in this study. (DOCX)

S3 Table Information and Genbank numbers of Galaxaura divaricata samples from various locations in the lagoon of Dongsha Atoll that were used for DNA barcoding in this study. (DOCX)

S4 Table Model fit statistics of percent cover of benthic categories (corals, total macroalgae, CCA, and other substrate) for reef top and reef slope among 12 sites. (DOCX)

S5 Table Model fit statistics of Galaxaura divaricata percent cover for reef top and reef slope among 13 sites. (DOCX)

S6 Table Relative percent cover (% mean ± SD, n = 45) of Galaxaura divaricata on 13 patch reef sites in the lagoon of Dongsha Atoll, South China Sea. (DOCX)

S7 Table Paucity of macrobenthic invertebrates in the lagoon of Dongsha Atoll. (DOCX)

S1 Text Detailed information of statistical analyses and results. (DOCX)
