# Supplementary figures and images for: Filamentous calcareous alga provides substrate for coral-competitive macroalgae in the degraded lagoon of Dongsha Atoll, Taiwan
Source: PLoS One. 2019 May 16;14(5):e0200864. doi: 10.1371/journal.pone.0200864 (PMC6522048; doi:10.1371/journal.pone.0200864)

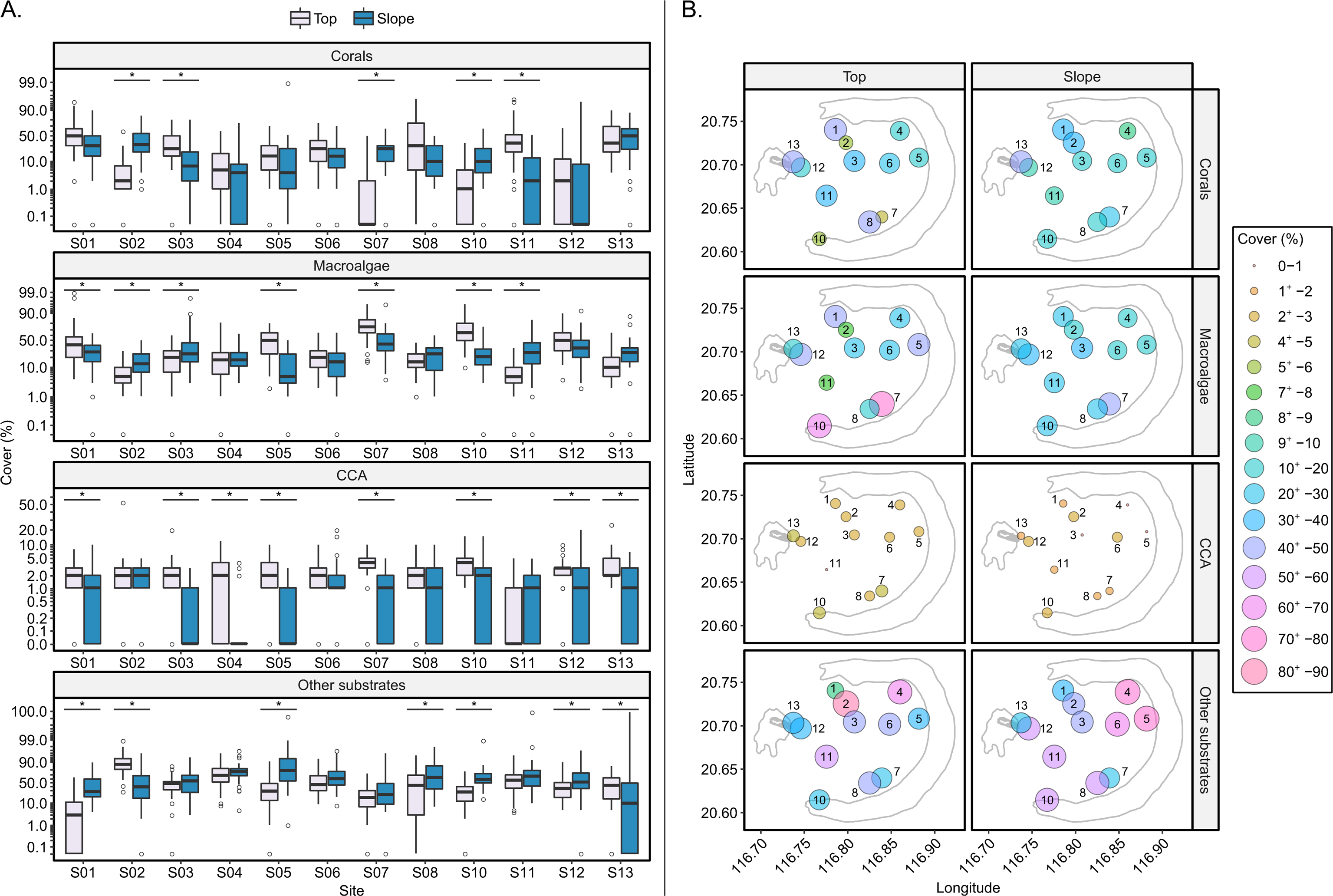

Supplement: S1 Fig — Percent cover (A) and spatial patterns (B) of corals, macroalgae, crustose coralline algae (CCA), and other substrate on reef top and slope across 13 sites. Color and size of the circles are proportional to the percent cover. (TIF) [file pone.0200864.s001.tif]

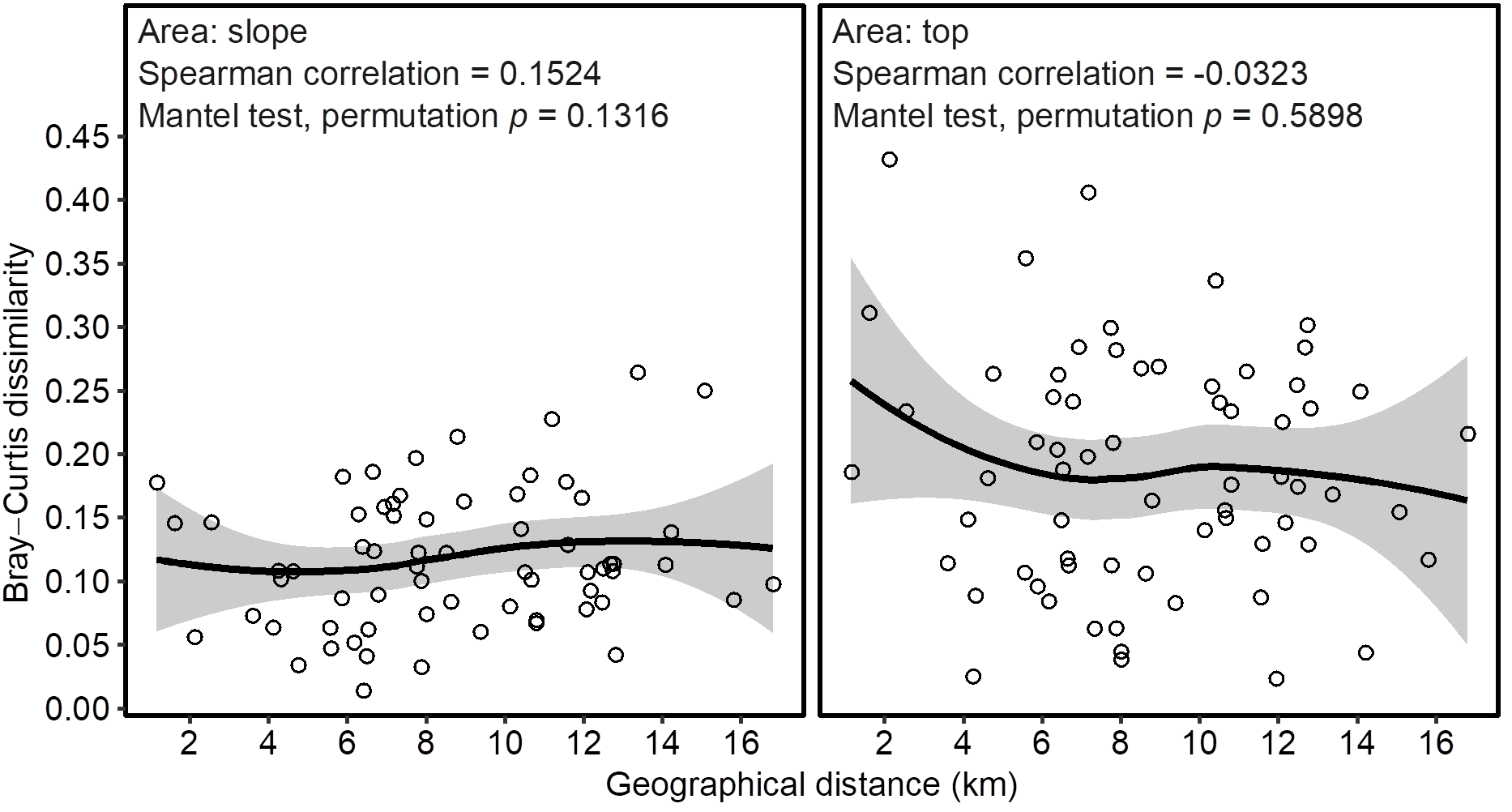

Supplement: S2 Fig — Regression lines and 95% confidence bands were estimated by the locally weighted scatterplot smoothing (LOESS) method. (TIF) [file pone.0200864.s002.tif]

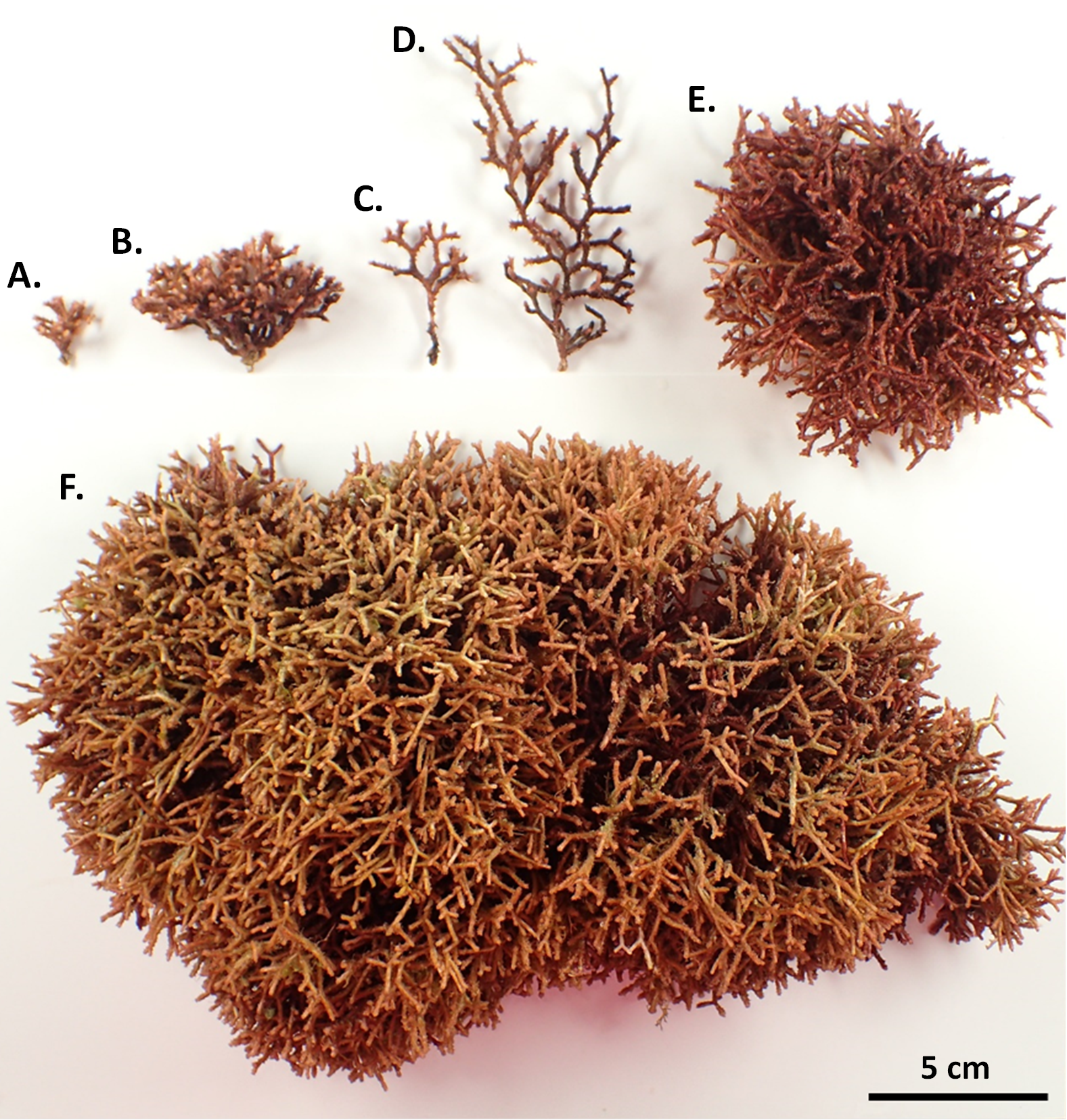

Supplement: S3 Fig — A-B) Small, ball-shaped thalli, and C-D) small, slender thalli were dominant on patch reefs in the north and northeast lagoon. E) Medium, ball-shaped thalli, and F) large carpet-like thalli were exclusively present in the southeast section of the lagoon. (TIF) [file pone.0200864.s003.tif]

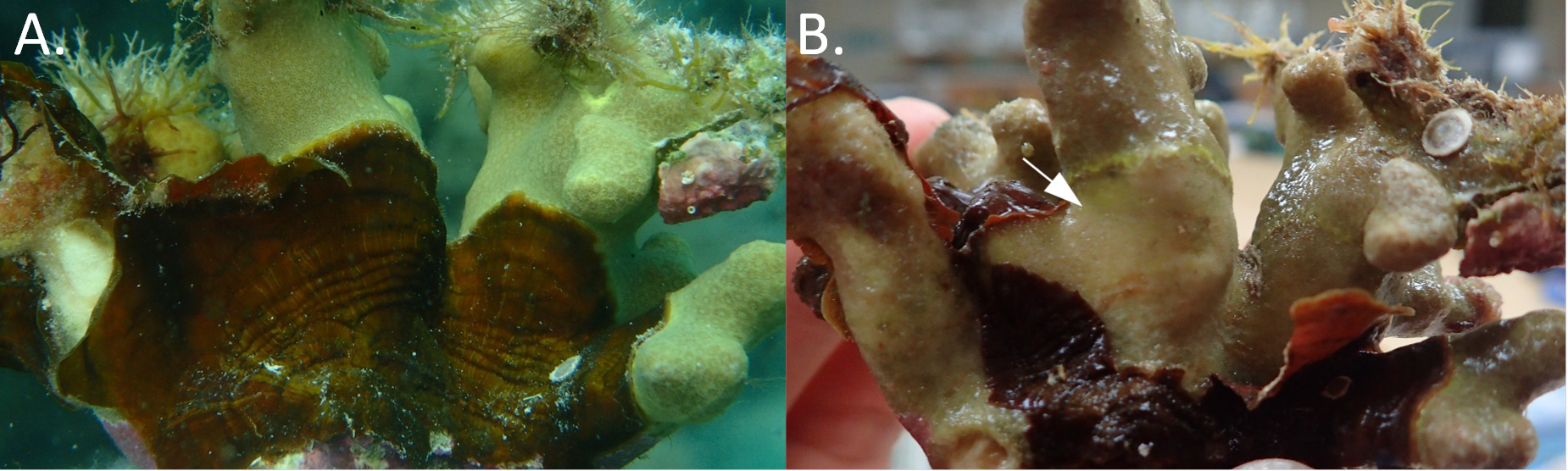

Supplement: S4 Fig — A) Coral overgrowth (Porites cylindrica in this case) by Lobophora sp28 is widely spread in the lagoon of Dongsha Atoll. B) The same coral showing dead tissue (arrow) after the removal of the algae. (TIF) [file pone.0200864.s004.tif]
