# Supplementary material for: Filamentous calcareous alga provides substrate for coral-competitive macroalgae in the degraded lagoon of Dongsha Atoll, Taiwan
Source: PLoS One. 2019 May 16;14(5):e0200864. doi: 10.1371/journal.pone.0200864 (PMC6522048; doi:10.1371/journal.pone.0200864)
Supplement: S1 Table — (DOCX) [file pone.0200864.s005.docx]

**S1 Table. GPS coordinates of patch reef survey sites in the lagoon of Dongsha Atoll, South China Sea (Taiwan).**

| Site | Latitude | Longitude |
| --- | --- | --- |
| 1 | 20^o^44'26.28'' | 116^o^47'8.879'' |
| 2 | 20^o^43'31.62'' | 116^o^47'52.679'' |
| 3 | 20^o^42'16.14'' | 116^o^48'27.419'' |
| 4 | 20^o^44'20.52'' | 116^o^51'35.699'' |
| 5 | 20^o^42'29.88'' | 116^o^52'54.599'' |
| 6 | 20^o^42'6.72'' | 116^o^50'53.279'' |
| 7 | 20^o^38'24'' | 116^o^50'20.999'' |
| 8 | 20^o^38'3.12'' | 116^o^49'30.479'' |
| 9 | 20^o^36'52.86'' | 116^o^49'24.179'' |
| 10 | 20^o^36'53.4'' | 116^o^46'2.399'' |
| 11 | 20^o^39'52.2'' | 116^o^46'32.159'' |
| 12 | 20^o^41'49.2'' | 116^o^44'45.659'' |
| 13 | 20^o^42'12.36'' | 116^o^44'14.639'' |
