# Supplementary material for: Filamentous calcareous alga provides substrate for coral-competitive macroalgae in the degraded lagoon of Dongsha Atoll, Taiwan
Source: PLoS One. 2019 May 16;14(5):e0200864. doi: 10.1371/journal.pone.0200864 (PMC6522048; doi:10.1371/journal.pone.0200864)
Supplement: S2 Table — (DOCX) [file pone.0200864.s006.docx]

**S2 Table.** **Information and Genbank numbers of macroalgae samples used for DNA barcoding in this study.**

| **Species** | **Phylum** | **Voucher#^a^** | **Date** | **Site** | **Area** | **Substrate** | **Marker** | **GenBank#** |
| --- | --- | --- | --- | --- | --- | --- | --- | --- |
| *Ceramium dawsonii* | Red | KO208 | Oct-16 | 7 | slope | *G. divaricata* | *rbc*L | MH048927 |
| *Coelothrix irregularis* | Red | K0206 | Oct-16 | 7 | slope | *G. divaricata* | *rbc*L | MH048928 |
| *Hypnea caespitosa* | Red | K0204 | Oct-16 | 7 | slope | *G. divaricata* | *rbc*L | MH048929 |
| *Hypnea caespitosa* | Red | K0203 | Oct-16 | 7 | slope | *G. divaricata* | *rbc*L | MH048930 |
| *Hypnea caespitosa* | Red | SD17120 | Aug-17 | 7 | slope | *G. divaricata* | *rbc*L | MH048931 |
| *Hypnea* sp. | Red | K0205 | Oct-16 | 7 | slope | *G. divaricata* | *rbc*L | MH048932 |
| *Lobophora* sp28^b^ | Brown | SD17023 | Aug-17 | 7 | slope | *G. divaricata* | *rbc*L | MH048934 |
| *Lobophora* sp28 | Brown | SD17021 | Aug-17 | 7 | slope | *G. divaricata* | *rbc*L | MH048935 |
| *Lobophora* sp28 | Brown | SD17019 | Aug-17 | 7 | slope | *G. divaricata* | *rbc*L | MH048936 |
| *Lobophora* sp28 | Brown | SD17017 | Aug-17 | 7 | slope | *G. divaricata* | *rbc*L | MH048937 |
| *Lobophora* sp28 | Brown | K0173 | Apr-16 | 7 | slope | Coral | *rbc*L | MH048940 |
| *Lobophora* sp28 | Brown | SD17058 | Aug-17 | 9 | slope | Coral | *rbc*L | MH048941 |
| *Padina* sp5^c^ | Brown | SD17114 | Aug-17 | 7 | slope | *G. divaricata* | *rbc*L | MH048933 |
| *Caulerpa chemnitzia* | Green | SD17117 | Aug-17 | 7 | slope | *G. divaricata* | *tufA* | MH048959 |

^a^Voucher specimens are deposited at the Tunghai University Herbarium (TUNG), Tunghai University, Taichung, Taiwan.

^b^Denomination according to [1].

^c^Denomination according to [2].
