# Supplementary material for: Filamentous calcareous alga provides substrate for coral-competitive macroalgae in the degraded lagoon of Dongsha Atoll, Taiwan
Source: PLoS One. 2019 May 16;14(5):e0200864. doi: 10.1371/journal.pone.0200864 (PMC6522048; doi:10.1371/journal.pone.0200864)
Supplement: S3 Table — (DOCX) [file pone.0200864.s007.docx]

**S3 Table. Information and Genbank numbers of *Galaxaura divaricata* samples from various locations in the lagoon of Dongsha Atoll that were used for DNA barcoding in this study.**

| **Species** | **Voucher#^a^** | **Date** | **Site** | **Area** | **Substrate** | **Thallus size** | **Marker** | **GenBank#** |
| --- | --- | --- | --- | --- | --- | --- | --- | --- |
| *G. divaricata* | K0210 | Apr-16 | 7 | slope | rock | medium | *rbc*L | MH048946 |
| *G. divaricata* | R90B12 | Feb-14 | 9 | top | rock | large | *rbc*L | MH048942 |
| *G. divaricata* | SD17048 | Aug-17 | 9 | slope | rubble | large | *rbc*L | MH048957 |
| *G. divaricata* | SD17098 | Aug-17 | 6 | top | rock | small | *rbc*L | MH048943 |
| *G. divaricata* | SD17099 | Aug-17 | 1 | top | rock | small | *rbc*L | MH048956 |
| *G. divaricata* | SD17100 | Aug-17 | 5 | top | rock | small | *rbc*L | MH048955 |
| *G. divaricata* | SD17101 | Aug-17 | 5 | top | rock | small | *rbc*L | MH048958 |
| *G. divaricata* | SD17102 | Aug-17 | 5 | slope | rock | medium | *rbc*L | MH048954 |
| *G. divaricata* | SD17103 | Aug-17 | 1 | slope | rock | small | *rbc*L | MH048953 |
| *G. divaricata* | SD17104 | Aug-17 | 6 | slope | rock | medium | *rbc*L | MH048952 |
| *G. divaricata* | SD17105 | Aug-17 | 5 | slope | rock | medium | *rbc*L | MH048951 |
| *G. divaricata* | SD17106 | Aug-17 | 6 | slope | rock | small | *rbc*L | MH048950 |
| *G. divaricata* | SD17107 | Aug-17 | 6 | slope | rock | small | *rbc*L | MH048944 |
| *G. divaricata* | SD17110 | Aug-17 | 6 | top | coral | medium | *rbc*L | MH048949 |
| *G. divaricata* | SD17112 | Aug-17 | 4 | slope | rock | medium | *rbc*L | MH048948 |
| *G. divaricata* | SD17113 | Aug-17 | 4 | slope | rock | small | *rbc*L | MH048947 |

^a^Voucher specimens are deposited at the Tunghai University Herbarium (TUNG), Tunghai University, Taichung, Taiwan.
