# Supplementary material for: Filamentous calcareous alga provides substrate for coral-competitive macroalgae in the degraded lagoon of Dongsha Atoll, Taiwan
Source: PLoS One. 2019 May 16;14(5):e0200864. doi: 10.1371/journal.pone.0200864 (PMC6522048; doi:10.1371/journal.pone.0200864)
Supplement: S4 Table — (DOCX) [file pone.0200864.s008.docx]

**S4 Table.** **Model fit statistics of percent cover of benthic categories (corals, total macroalgae, CCA, and other substrate) for reef top and reef slope among 12 sites.**

| Model | Independent variable | | |  | Bayes *R*^2^ average | | | | LOOIC^a^ | Posterior probability^b^ | Bayes factor | | | |
| --- | --- | --- | --- | --- | --- | --- | --- | --- | --- | --- | --- | --- | --- | --- |
|  | Area (fixed) | Random slope against area by site | Random intercept by site |  | Corals | Macro-algae | CCA | Other substrate |  |  | Over M_1_ | Over M_2_ | Over M_3_ | Over M_4_ |
| M_1_ | Yes | Yes | Yes |  | 0.322 | 0.335 | 0.248 | 0.314 | 16474.3 | < 0.001 | 1 | **< 0.001** | < 0.001 | < 0.001 |
| M_2_ | No | Yes | Yes |  | 0.340 | 0.379 | 0.204 | 0.331 | 6246.3 | 0.999 | > 150 | 1 | **> 150** | > 150 |
| M_3_ | No | No | Yes |  | 0.197 | 0.232 | 0.070 | 0.195 | 6954.0 | < 0.001 | < 0.001 | < 0.001 | 1 | **> 150** |
| M_4_ | No | No | No |  | 0 | 0 | 0 | 0 | 7575.8 | < 0.001 | > 150 | < 0.001 | < 0.001 | 1 |

^a^LOOIC denotes leave-one-out cross-validation information criterion. Lower value indicates a better model fitting in comparison to other models.

^b^Sum of all posterior probabilities is constrained to 1. Higher values indicates a better model fitting in comparison to other models.
