# Supplementary material for: Filamentous calcareous alga provides substrate for coral-competitive macroalgae in the degraded lagoon of Dongsha Atoll, Taiwan
Source: PLoS One. 2019 May 16;14(5):e0200864. doi: 10.1371/journal.pone.0200864 (PMC6522048; doi:10.1371/journal.pone.0200864)
Supplement: S5 Table — (DOCX) [file pone.0200864.s009.docx]

**S5 Table.** **Model fit statistics of G. divaricata percent cover for reef top and reef slope among 13 sites.**

| Model | Independent variable | | | Bayes *R*^2^ average | LOOIC^a^ | Posterior probability^b^ | Bayes factor | | | |
| --- | --- | --- | --- | --- | --- | --- | --- | --- | --- | --- |
|  | Area (fixed) | Random slope against area by site | Random intercept by site |  |  |  | Over M_1_ | Over M_2_ | Over M_3_ | Over M_4_ |
| M_1_ | Yes | Yes | Yes | 0.758 | 3782.02 | 0.116 | 1 | **0.131** | > 150 | > 150 |
| M_2_ | No | Yes | Yes | 0.758 | 3781.72 | 0.884 | 7.634 | 1 | **> 150** | > 150 |
| M_3_ | No | No | Yes | 0.640 | 4232.28 | < 0.001 | < 0.001 | < 0.001 | 1 | > 150 |
| M_4_ | No | No | No | 0 | 5415.85 | < 0.001 | < 0.001 | < 0.001 | <0.001 | 1 |

^a^LOOIC denotes leave-one-out cross-validation information criterion. Lower value indicates a better model fitting in comparison to other models.

^b^Sum of all posterior probabilities is constrained to 1. Higher values indicates a better model fitting in comparison to other models.
