# Supplementary material for: Filamentous calcareous alga provides substrate for coral-competitive macroalgae in the degraded lagoon of Dongsha Atoll, Taiwan
Source: PLoS One. 2019 May 16;14(5):e0200864. doi: 10.1371/journal.pone.0200864 (PMC6522048; doi:10.1371/journal.pone.0200864)
Supplement: S6 Table — (DOCX) [file pone.0200864.s010.docx]

**S6 Table. Relative percent cover (% mean ± SD, n= 45) of *Galaxaura divaricata* on 13 patch reef sites in the lagoon of Dongsha Atoll, South China Sea.**

| **Site** | **Location** | **Patch reef area** | |
| --- | --- | --- | --- |
|  |  | **Top (1-5 m)** | **Slope (5-10 m)** |
| 1 | North | 0.02 + 0.08 | 0.02 + 0.15 |
| 2 | North | 0 | 0 |
| 3 | North | 0.27 + 0.78 | 0.31 + 0.9 |
| 4 | Northeast | 1.52 + 1.9 | 1.47 + 1.95 |
| 5 | Northeast | 5.69 + 5.47 | 3.37 + 5.62 |
| 6 | Northeast | 0.79 + 1.36 | 0.21 + 0.34 |
| 7 | Southeast | 0.02 + 0.15 | 15.86 + 17 |
| 8 | Southeast | 0.17 + 0.67 | 4.31 + 6.67 |
| 9 | Southeast | 41.87 + 24.73 | 40.87 + 25.58 |
| 10 | South | 0 | 0 |
| 11 | Center | 0.46 + 1.41 | 0.02 + 0.15 |
| 12 | West | 0 | 0 |
| 13 | West | 0 | 0 |
