# Supplementary material for: Filamentous calcareous alga provides substrate for coral-competitive macroalgae in the degraded lagoon of Dongsha Atoll, Taiwan
Source: PLoS One. 2019 May 16;14(5):e0200864. doi: 10.1371/journal.pone.0200864 (PMC6522048; doi:10.1371/journal.pone.0200864)
Supplement: S7 Table — (DOCX) [file pone.0200864.s011.docx]

**S7 Table.** **Paucity of macrobenthic invertebrates in the Dongsha lagoon.**

| **Macrobenthic fauna** | **Count** | **Density (individual/100 m^2^)** |
| --- | --- | --- |
| *Diadema savignyi* | 3 | 0.03 |
| *Diadema setosum* | 1 | 0.01 |
| *Echinometra mathaei* | 126 | 1.26 |
| *Echinothrix calamaris* | 3 | 0.03 |
| *Tripneustes gratilla* | 4 | 0.04 |
| *Culcita novaeguineae* | 54 | 0.54 |
| *Echinaster luzonicus* | 2 | 0.02 |
| *Fromia* spp. | 8 | 0.08 |
| *Linckia multifora* | 15 | 0.15 |
| *Holothuria* | 0 | 0 |
| *Cypraea tigris* | 3 | 0.03 |
| Giant clam | 34 | 0.34 |
| *Lambis* spp. | 3 | 0.03 |
| Lobster | 0 | 0 |

Data derived from a belt transect survey of 13 patch reefs and seven seagrass beds (10,000 m^2^ total area surveyed) across the lagoon of Dongsha Atoll in September 2017.
