# Supplementary material for: Filamentous calcareous alga provides substrate for coral-competitive macroalgae in the degraded lagoon of Dongsha Atoll, Taiwan
Source: PLoS One. 2019 May 16;14(5):e0200864. doi: 10.1371/journal.pone.0200864 (PMC6522048; doi:10.1371/journal.pone.0200864)
Supplement: S1 Text — (DOCX) [file pone.0200864.s012.docx]

**S1 Text. Detailed information of statistical analyses and results.**

**Materials and Methods**

The main reasons fitting cover data by using Bayesian MCMC approaches rather than traditional frequentist approaches (e.g. frequentist MLE or permutation RDA) including that 1) MLE failed to estimate the random effects (no convergence) and 2) very difficult to take account of both random slope and random intercept with RDA. Furthermore, Bayesian MCMC approach allowed us to perform multivariate and univariate analysis simultaneously and to deal with multiple comparisons after univariate/multivariate analysis with controlling false discover rates. We assigned weak informative priors for all parameters (intercept term, 10 times scaled T distribution with DF = 3; fixed effect, 5 times scaled T distribution with DF = 7; random effect and residual, exponential distribution with rate = 1). 6,000 MCMC iterations (including the beginning 5,000 burn-in iterations) per thread were performed, and a total of 10 parallel threads were parallel proceeded for each parameter. Strength of evidence of a parameter was evaluated by Bayes factor (BF) **between full model and reduced model. Partial *R*^2^ of a dependent variable was sequentially determined by the average Bayes *R*^2^ increase between full model and that of reduced model.**
